# Integrative analysis of DNA copy number and gene expression in metastatic oral squamous cell carcinoma identifies genes associated with poor survival

**DOI:** 10.1186/1476-4598-9-143

**Published:** 2010-06-11

**Authors:** Chang Xu, Yan Liu, Pei Wang, Wenhong Fan, Tessa C Rue, Melissa P Upton, John R Houck, Pawadee Lohavanichbutr, David R Doody, Neal D Futran, Lue Ping Zhao, Stephen M Schwartz, Chu Chen, Eduardo Méndez

**Affiliations:** 1 Department of Otolaryngology-Head and Neck Surgery, University of Washington, Seattle, WA 98195, USA; 2 Program in Biostatistics & Biomathematics, Public Health Sciences Division, Fred Hutchinson Cancer Research Center, Seattle, WA 98109, USA; 3 Program in Cancer Prevention and Biostatistics, Public Health Sciences Division, Fred Hutchinson Cancer Research Center, Seattle, WA 98109, USA; 4 Department of Biostatistics, University of Washington, Seattle, WA 98195, USA; 5 Department of Pathology, University of Washington, Seattle, WA 98195, USA; 6 Program in Epidemiology, Public Health Sciences Division, Fred Hutchinson Cancer Research Center, Seattle, WA 98109, USA; 7 Department of Epidemiology, University of Washington, Seattle, WA 98195, USA; 8 Clinical Research Division, Fred Hutchinson Cancer Research Center, Seattle, WA 98109, USA; 9 Surgery and Perioperative Care Service, VA Puget Sound Health Care System, Seattle, Washington 98108, USA

## Abstract

**Background:**

Lymphotropism in oral squamous cell carcinoma (OSCC) is one of the most important prognostic factors of 5-year survival. In an effort to identify genes that may be responsible for the initiation of OSCC lymphotropism, we examined DNA copy number gains and losses and corresponding gene expression changes from tumor cells in metastatic lymph nodes of patients with OSCC.

**Results:**

We performed integrative analysis of DNA copy number alterations (CNA) and corresponding mRNA expression from OSCC cells isolated from metastatic lymph nodes of 20 patients using Affymetrix 250 K Nsp I SNP and U133 Plus 2.0 arrays, respectively. Overall, genome CNA accounted for expression changes in 31% of the transcripts studied. Genome region 11q13.2-11q13.3 shows the highest correlation between DNA CNA and expression. With a false discovery rate < 1%, 530 transcripts (461 genes) demonstrated a correlation between CNA and expression. Among these, we found two subsets that were significantly associated with OSCC (n = 122) when compared to controls, and with survival (n = 27), as tested using an independent dataset with genome-wide expression profiles for 148 primary OSCC and 45 normal oral mucosa. We fit Cox models to calculate a principal component analysis-derived risk-score for these two gene sets ('122-' or '27-transcript PC'). The models combining the 122- or 27-transcript PC with stage outperformed the model using stage alone in terms of the Area Under the Curve (AUC = 0.82 or 0.86 vs. 0.72, with *p *= 0.044 or 0.011, respectively).

**Conclusions:**

Genes exhibiting CNA-correlated expression may have biological impact on carcinogenesis and cancer progression in OSCC. Determination of copy number-associated transcripts associated with clinical outcomes in tumor cells with an aggressive phenotype (i.e., cells metastasized to the lymph nodes) can help prioritize candidate transcripts from high-throughput data for further studies.

## Background

Oral squamous cell carcinoma (OSCC) is the sixth most common cancer worldwide. The presence of lymph node metastasis is associated with a 50% decrease in 5-yr survival, and is the single most important prognostic factor identified to date [1-4]. However, the mechanisms by which OSCC cells spread from the primary site to local lymph nodes is not well understood. Transcriptome profiling has been used to gain insights into this process [1,5-7], but the function of many of the proposed differentially expressed transcripts is unknown. To improve the likelihood of finding genes driving the carcinogenic process, several groups have exploited the common feature of genomic instability in cancer [8] and identified genes the expression of which is correlated with corresponding DNA copy number in tumors such as brain, breast, ovarian, liver, multiple myeloma, and melanoma [9-18].

In an attempt to identify novel driver genes responsible for the OSCC metastasis, we utilized a recently developed protocol by our group for high-throughput profiling of DNA and RNA from the same cell population obtained by laser capture microdissection (LCM) to determine the association between DNA copy number aberration (CNA) and gene expression in tumor cells isolated from metastatic lymph nodes. We reasoned that these cells would contain those changes in the genome and transcriptome that are essential to the lymphotropism of OSCC. In addition, we tested the hypothesis that since nodal metastases are associated with poor prognosis, the expression of copy number-associated genes from metastatic OSCC tumor cells is associated with survival.

## Results

### Study population

Selected characteristics of the 20 OSCC patients with lymph node metastases are shown in Additional file 1, Table S1. Eight patients had cancers arising in the oropharynx whereas the remainder of the tumors arose from the oral cavity. The age range was 23-84 (mean 56.8) years. With the exception of three patients with only one positive lymph node, the majority of patients had ≥ N2 nodal staging (i.e. multiple metastatic nodes detected).

### DNA copy number aberrations in OSCC nodal metastasis

CNA events were detected in all of the 20 OSCC lymph node metastases and in all chromosomal arms that were covered with SNP probes (13p, 14p, 15p, 21p and 22p were not covered by the Affymetrix 250 K Nsp SNP array). The percentage of each genome showing CNA ranged between 25.6% - 73.9% (mean ± sd: 48.7 ± 13.6%. CNA was defined as the ratio of DNA copy number in cancer cells vs. normal cells either < 0.93 or > 1.07. In particular, large regions of amplification were detected on chromosome arms 3q, 5p, 8q, and 9q and large regions of deletion were detected on chromosome arms 3p, 5q, 8p, and 13q. Most of the previously reported CNA that have previously been associated with poor outcome in patients with OSCC or head and neck squamous cell carcinoma [19] were detected in the samples, such as amplification in regions 3q21-29, 5p15, 7p12, 8q21-24, 11q13, 12q24, 14q23-32, 16q22 and 20q, and deletion in regions 1p21, 5q11-12, 5q14-15, 5q31, 8p21-22, 9p21, 10p12, 11q23-25, 12q22, 18q11.2, 21q and 22q (see Additional file 2, Figure S1).

### Inferring genome DNA copy number for each transcript

Out of the 54,675 transcripts measured by the Affymetrix U133 Plus 2.0 array, 23,484 transcripts (43.0%) remained after filtering as described in the Methods. However, the majority of these transcripts did not overlap with a SNP that could be used for inferring the DNA copy number (Additional file 3, Table S2). Therefore, we incorporated the SNPs within a 250 kb upstream and downstream of neighboring region for each transcript. For 18 transcripts there were no SNPs within the 250 kb neighboring region, leaving 23,466 transcripts for determining the relationship between DNA copy number and gene expression. Out of these, the DNA copy number was inferred from at least 5 SNPs for 23,319 transcripts (99.4% of the 23,466 transcripts) and from 1-4 SNPs, for 147 transcripts (Additional file 3, Table S2).

### Association of DNA copy number on gene expression

**Figure 1:**
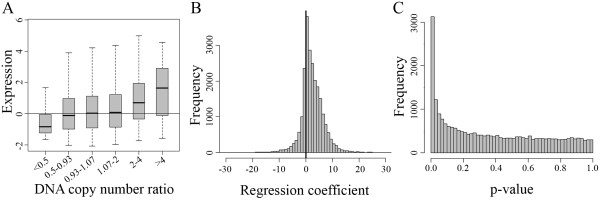
**Influence of DNA copy number on gene expression in the 20 lymph node metastatic OSCC**. A. Summary of gene expression in subgroup of transcripts with different cancer-normal DNA copy number ratio (Box plot in each subgroup indicates 25^th^, 50^th^, and 75^th ^percentile of the gene expression). X-axis: subgroups with different DNA copy number ratio. Y-axis: base-2 log-transformed relative gene expression; B. Distribution of the estimated correlation coefficients between DNA copy number and gene expression. X-axis: value of the correlation coefficient; Y-axis: frequency; C. P-value distribution for the correlation coefficients.

Based on the distribution of the cancer-normal DNA copy number ratio, the 23,466 transcripts were divided into six subgroups, representing high level deletion (ratio < 0.5), low level deletion (ratio: 0.5-0.93), no change (ratio: 0.93-1.07), low- (ratio: 1.07-2), medium- (ratio: 2-4), and high-level amplification (ratio > 4), respectively. The average gene expression in the low level genome amplification and deletion subgroups differed little from that in the no change subgroup (Figure 1A). However, the average gene expression in the high level deletion subgroup was -0.8 (base-2 log-transformed, which is equivalent to 1.7-fold lower) and the average gene expression in the medium and high level amplification subgroups was 0.7 and 1.6 (equivalent to 1.6-fold and 3.0-fold higher), respectively, as compared to that in the no change subgroup (Figure 1A). Thus, there was a dose-response relationship between DNA copy number and gene expression (Figure 1A).

To determine the relationship between DNA copy number and individual gene expression, we applied a robust linear regression model. The distribution of the correlation coefficients forms a normal-shaped curve with the mean of the distribution shifted to the positive direction (Figure 1B), indicating a large set of the genes having positive correlations between DNA copy number and gene expression. Based on the p-value distribution, we estimated that 30.6% of the transcripts showed statistically significant correlations between DNA copy number and expression (Figure 1C). With a False Discovery Rate (FDR) < 1%, we identified 530 transcripts (461 genes) with a significantly correlated DNA copy number and expression.

### Genome region 11q13.2-q13.3 shows the highest correlation between DNA CNA and expression in metastatic OSCC

**Figure 2:**
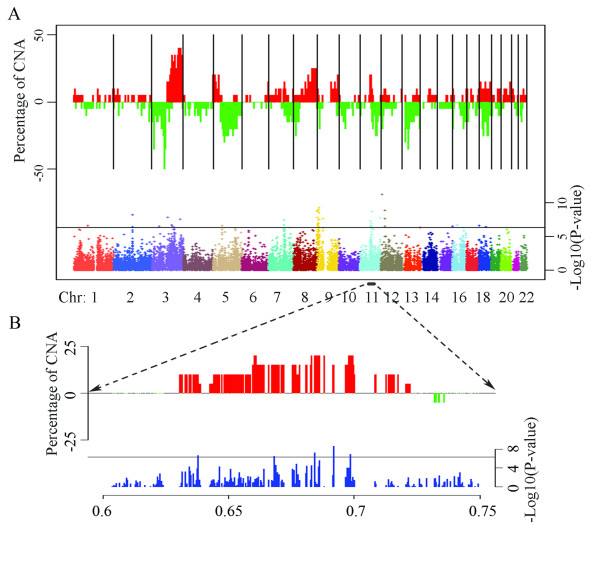
**Summary of genome-wide DNA copy number aberrations and concordant gene expression changes**. A. Top panel: Percentage of genome DNA amplification (red) and deletion (green) of the 20 OSCC on the human autosomes. Only CNAs with tumor-normal DNA copy number ratio less than 0.7 or greater than 1.4 are counted as deleted or amplified, respectively. Bottom-panel: Manhattan plot of the negative base-10 log-transformed p-value for the correlation coefficients between DNA copy number and gene expression for all the studied genes at each loci. Line indicates the Bonferroni p-value cutoff = -log10(0.01/23,466) = 6.37; B. A close-up view of the 11q13.2-q13.3 high correlation region.

**Figure 3:**
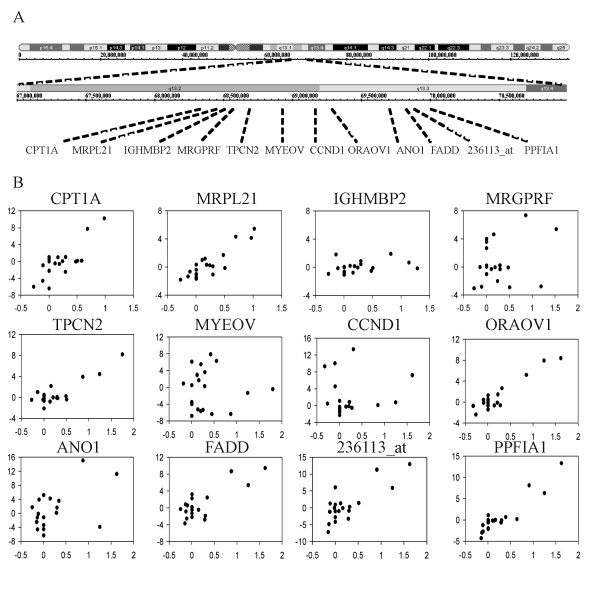
**Correlation between DNA copy number and gene expression for genes in the high correlation region of chromosome 11q13.2-q13.3**. A. Locations of the genes and the unknown transcript in the region. B. DNA copy number (x-axis, base-2 log-transformed ratio of cancer to normal) and gene expression (y-axis, base-2 log-transformed intensity) of each gene in the 20 lymph node metastatic OSCC. For genes measured by multiple probe sets, the probe set with the strongest correlation is shown.

Although we observed significant associations between gene expression and CNA on all chromosomal arms covered with SNP probes (Figure 2A), chromosomal region 11q13.2-q13.3 contained a cluster of genes with significantly high correlation between DNA copy number and gene expression (i.e. *p *< 4.26 × 10-7, Bonferroni correction value) (Figure 2B). We further examined the genes in the region as previous studies had associated DNA amplification in this region with poor prognosis in OSCC patients [20]. There were 20 probe sets in this region that measured the expression of 12 genes (Figure 3A). Seven of these genes, *CPT1A, MRPL21, TPCN2, ORAOV1, FADD*, and *PPFIA1*, and an unknown transcript measured by the probe set 236113_at, showed high correlation between DNA copy number and gene expression. The remaining five genes in the same region, *IGHMBP2, MRGPRF, MYEOV, CCND1*, and *ANO1*, showed no significant correlation between DNA copy number and gene expression (Figure 3B).

### Copy number-associated transcripts can distinguish OSCC from normal oral mucosa and are associated with survival

To prioritize the 530 transcripts whose CNA and expression are significantly correlated, we examined their relationship with OSCC (vs. normal oral mucosa from control individuals) and with survival. We used a previously reported dataset containing transcriptome profiles for 167 OSCC primary tumors and 45 controls [21]. The baseline characteristics of the OSCC patients included in the survival analysis were presented previously in Méndez et al (Additional file 1, Table S1) [22]. Nineteen of the 20 patients who contributed lymph nodes for this study were also part of the 167 patients in the testing dataset. They were therefore excluded from further analysis. From the remaining 148 cases, 132 had at least 4 months of follow-up, a cut-off used to reduce the inclusion of deaths from patients due to co-morbidities rather than tumor biology. The range of follow-up time for these patients known to be alive from initial enrollment (December 16, 2003) to the date of last follow-up (April 17, 2007) was 4.4 to 38.7 months, with a median of 17.0 months. Thirty six patients had died by the end of the follow-up period; there were 25 OSCC-specific deaths, nine non-OSCC-specific deaths and two deaths of unknown causes. To test the hypothesis that these 530 transcripts were associated with survival, we estimated hazard ratios (HR) for OSCC-specific mortality, adjusting for age and sex. Although the expression profiles in this dataset were generated using primary tumors and the DNA copy number-associated transcripts identified in this study was based on lymph node metastases, we hypothesized some of the changes have already happened in the primary tumors. The selected 530 transcripts were significantly enriched with genes differentially expressed in OSCC cases versus controls (*p *< 0.001) when compared with random gene sets of the same size. The list was also significantly enriched with genes associated with survival (*p *< 0.001). With the same cut-off value of Z-score > 6 as in our previous study [21], 122 transcripts representing 116 genes were identified as differentially expressed in OSCC cases versus controls (Additional file 4, Table S3). Ingenuity Pathways Analysis (IPA v8.5, Ingenuity^® ^Systems, http://www.ingenuity.com was able to functionally categorize 88 of the 116 genes, of which the following molecular and cellular functional categories with the largest number of genes involved: "Gene Expression" (25 genes: *ACTL6A, ATF7, BAG1, CCND2, EIF2S1, EN1, ERCC5, FADD, GTF2E1, HLTF, MBD2, MCM7, MUC1, MYNN, NFX1, OSMR, PAK2, PBX1, RAF1, RBBP8, SLU7, SNAPC3, TAF6, VAV3*, and *ZHX1*), "Cell Cycle" (15 genes: *ACTL6A, CCND2, CSNK2A2, FADD, MCM7, MPHOSPH6, PAK2, PCM1, PPP2R2A, RAF1, RBBP8, SENP5 *(includes EG:205564), *SSSCA1, TAF6*, and *VAV3*), and "Cell Death" (12 genes: *ATF7, BAG1, CCND2, CSNK2A2, EN1, FADD, MUC1, PAK2, PBX1, PPP2R2A, RAF1*, and *TAF6*). In addition, 27 transcripts representing 24 genes were associated with survival (Table 1). IPA was able to functionally categorize 19 of the 24 genes, of which the following molecular and cellular functional categories with the largest number of genes involved: "Cell Death" (*CCT6A, FADD, NFE2L2, RAD23B, STEAP3*, and *TREM1*) and "Cellular Movement" (*FADD, NFE2L2*, and *PPFIA1*).

**Table 1:** Transcripts associated with survival

Gene	Gene Title	By Probe Set	Note*
C7orf30	chromosome 7 open reading frame 30	226386_at	OSCC associated
C8orf59	chromosome 8 open reading frame 59	1555241_at	OSCC associated
C8orf76	chromosome 8 open reading frame 76	225702_at	
CCT6A	chaperonin containing TCP1, subunit 6A (zeta 1)	201327_s_at	OSCC associated
DCAF13	DDB1 and CUL4 associated factor 13	225676_s_at	OSCC associated
DNAJC8	DnaJ (Hsp40) homolog, subfamily C, member 8	212490_at	OSCC associated
FADD	Fas (TNFRSF6)-associated via death domain	202535_at	OSCC associated
FAF2	Fas associated factor family member 2	212108_at	OSCC associated
FUBP3	far upstream element (FUSE) binding protein 3	212824_at	
MRPL15	mitochondrial ribosomal protein L15	218027_at	OSCC associated
NFE2L2	nuclear factor (erythroid-derived 2)-like 2	201146_at	OSCC associated
OSMR	oncostatin M receptor	1554008_at	OSCC associated
PPFIA1	protein tyrosine phosphatase, receptor type, f polypeptide (PTPRF), interacting protein (liprin), alpha 1	210235_s_at; 202066_at; 210236_at	OSCC associated
PURB	purine-rich element binding protein B	227718_at	OSCC associated
RAD23B	RAD23 homolog B (S. cerevisiae)	201223_s_at	
RANBP6	RAN binding protein 6	213019_at	
SSSCA1	Sjogren syndrome/scleroderma autoantigen 1	203114_at	OSCC associated
STEAP3	STEAP family member 3	218424_s_at; 1554830_a_at	OSCC associated
TOR1B	torsin family 1, member B (torsin B)	209593_s_at	OSCC associated
TREM1	triggering receptor expressed on myeloid cells 1	219434_at	OSCC associated
UCK2	uridine-cytidine kinase 2	209825_s_at	OSCC associated
VPS54	vacuolar protein sorting 54 homolog (S. cerevisiae)	222627_at	OSCC associated
YIPF5	Yip1 domain family, member 5	224934_at	
ZNF410	zinc finger protein 410	209944_at	

*OSCC associated: gene is differentially expressed in OSCC vs. normal controls.

**Figure 4:**
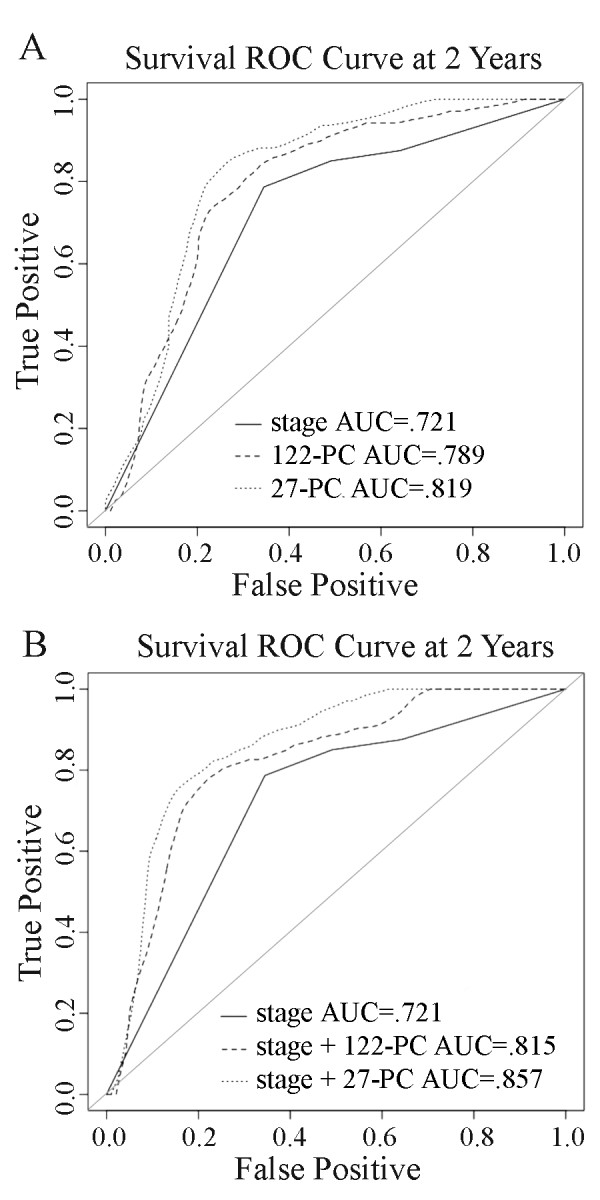
**ROC analysis of 2-year survival comparing AJCC stage with gene expression data**. A, ROC curves for 2-year survival for models "stage," "122-PC," and "27-PC" B, ROC curves for 2-year survival for models "stage," "stage + 122-PC" and "stage + 27-PC".

Given results from our previous study showing that genes that are differentially expressed between OSCC and normal oral mucosa are associated with OSCC prognosis [22], we tested whether gene expression levels of the 122 transcripts were associated with survival. We used the first two principal component (PC) scores of the 122 transcripts as a predictor to fit Cox models. We also tested a model using the first two PC scores of the 27 survival-associated transcripts. These two models were compared with AJCC stage alone, a current clinical guideline for survival prediction. The hazard ratios for five models with either stage or PC scores or with a combination of stage and PC scores are summarized in Table 2. Results from the ROC analysis for these five models are shown in Figure 4. The AUC for a model combining stage and the 122- or 27-transcript PC risk scores was significantly higher than for a model with stage alone (*p *= 0.044 and 0.011, respectively) (Figure 4A and 4B).

**Table 2:** Hazard ratios association of AJCC stage and gene expression with survival

Model terms	Hazard Ratio (95% CI*)		
	
	Unadjusted**	122-gene PC + stage	27-gene PC + stage
AJCC stage	2.24 (1.35-3.71)	2.13 (1.29-3.54)	2.04 (1.24-3.35)
122-gene 1st PC***	1.22 (1.1-1.35)	1.2 (1.08-1.34)	
122-gene 2nd PC	0.92 (0.83-1.03)	0.94 (0.84-1.04)	
27-gene 1st PC	1.28 (1.14-1.44)		1.24 (1.11-1.39)
27-gene 2nd PC	1.34 (1.07-1.69)		1.27 (1.01-1.6)

* CI: Confidence Interval

**The unadjusted HRs refer to 3 separate models: one for AJCC stage, one for the two 122-gene PCs, and one for the two 27-gene PCs

***PC: Principal Component.

## Discussion

In this study, we analyzed the relationship between DNA copy number and gene expression in tumor cells from metastatic lymph nodes of 20 OSCC patients to: 1) identify the genes showing a significant correlation between DNA copy number and gene expression, and 2) determine which if any copy number-associated genes from metastatic OSCC were associated with OSCC status and survival. We used the same cells to isolate and amplify DNA and RNA for high-throughput profiling whereas in previous studies, DNA and RNA were interrogated either from biopsy samples, or if tumor-cell specific, from two different laser microdissected cell populations. To our knowledge, this is the first time the genome and transcriptome of a solid tumor have been profiled and integrated using the genetic materials from the same cancer cell population enriched by LCM. This is also the first integrative analysis of DNA copy number and gene expression of tumor cells from metastatic OSCC lymph nodes.

There are a number of unresolved issues when interpreting data that integrate genome-wide DNA copy number and gene expression profiles. Study design, statistical analyses and quality of specimens are all factors that can all impact in how inferences are drawn between DNA copy number and gene expression. An early study in breast cancer indicated that at least 12% of all the variance in gene expression could be directly attributable to the underlying variation in DNA copy number [11]. As the authors pointed out, however, that was a significant underestimate as DNA was obtained from tissue biopsies with mixed tumor and stromal cells, reducing the ability to detect tumor-associated CNA. In a more recent study of non-small-cell lung carcinoma, 42% of the genes were classified as DNA copy number-driven [23]. However, the study did not enrich for tumor cells and dichotomized genome CNA as loss and gain only, thereby limiting the assessment of DNA copy number's impact on expression. Another breast cancer study that enriched the tumor cells via microdissection detected 46.7% of genes with a significant correlation between copy number and gene expression. However, that study did not appear to have adjusted for multiple comparisons and this may lead to over-estimation of the impact of copy number on gene expression [24]. Regardless of these limitations, data from these studies and ours does point that, in general increased DNA copy number was associated with increased expression. A critical issue is how to distinguish those copy number-associated genes which are the true "drivers" of carcinogenesis from those without functional relevance. We believe that determining which copy number-associated transcripts are associated with clinical characteristics and survival could be one approach to prioritize these transcripts further.

One interesting finding is the complexity seen between DNA copy number and gene expression for individual CNA regions. In the same DNA region, it was not unusual to find a mix of both genes the expression of which did and did not show a correlation with CNA. For instance, six genes and an unknown transcript located in the high correlation 11q13.2-q13.3 region showed a strong positive correlation between DNA copy number and gene expression, whereas the expression of five other genes located in the same region can not be explained by their CNA. Previous studies have linked five of the six copy number-associated genes in the 11q13.2-q13.3 region to cancer progression, including: 1) the genome amplification and overexpression of *ORAOV1 *and *PPFIA1 *associated with aggressive phenotypes in OSCC cell lines [20,25]; 2) the expression of the cell apoptotic signal mediator *FADD *associated with nodal metastasis in non-small cell lung cancer [26] and poor survival in laryngeal carcinoma [27]; and 3) the expression of *CPT1A *and *MRPL21 *associated with the development of colon cancer and breast cancer, respectively [28,29]. Therefore, at least some associations between DNA copy number and gene expression can be shown to have clinical or pathogenetic relevance, as opposed to only representing a DNA dosage effect.

Many of the gene candidates identified in this study have mechanistic relevance in regards to how cancer cells migrate from the primary organ to distant sites and survive through this process and in their new environment. For example, out of the 116 OSCC specific genes, at least 20 are involved in either cell cycle or cell death. Similarly, for the 24 genes associated with survival, 6 genes are involved in cell death and 3 genes are involved in cell migration. The expression changes of these genes, which our study suggests occurs as a result of copy number alterations, may enhance the viability and migration capability of the cancer cells, thus assisting in the progression and spread of the OSCC. Further investigation of these genes to confirm their roles in OSCC metastasis is warranted.

Although genes with CNA-associated expression were located on all chromosomal arms studied, several clusters of such genes were observed (e.g., in the 11q13 region as described above and other regions such as 7q22.1 and 9p24.1 (see Figure 2)). Likely, a single CNA event in these regions would cause the dysregulated expression of multiple genes. It is generally believed that recurrent genome CNA regions harbour genes that are essential to cancer progression, but many current functional studies focus on identifying the single "key driver gene" in the region of CNA responsible for cancer progression. In a recent study on liver cancer, however, the over-expression of both *cIAP1 *and *Yap*, as a result of genome amplification at mouse chromosome 9qA1, cooperatively promoted tumorigenesis [16]. In another study of glioblastoma, chromosome 12q13.3-14.1 amplification caused over-expression of genes (*CDK4 *and *CENTG1*) and a microRNA (*hsa-miR-26a*), all of which were demonstrated to contribute to the progression of the cancer [30]. Thus, CNA in these regions may be an efficient mechanism for cancer cells to obtain functional benefits from multiple genes (and even microRNAs) to achieve a specific aberrant capability. Likewise, amplification at 11q13 is one of the most frequently observed CNA events in almost all solid tumors [31]. This single CNA event would likely cause the over-expression of genes such as *ORAOV1*, *PPFIA1*, and *FADD*, which, as mentioned above would synergize to promote OSCC progression. For this reason, genes with copy number associated expression in the regions of CNA reported in this study should be considered in a comprehensive way as concurrent dysregulation of these genes could contribute to the lymphotropic phenotype of these metastatic tumor cells.

The fact that among CNA-associated genes found in tumor cells isolated from nodal metastases, we found subsets that were significantly associated with OSCC status and survival using expression profiles of biopsy samples from primary tumors which were not laser-dissected suggests that the signal from our copy number-associated genes was robust enough to overcome "noise" from the expression signal of bystander cells. Moreover, the fact that the two survival models incorporating the gene expression of the '122- or 27-transcript PC' resulted in higher AUCs than a model with stage alone suggests that at least some of these copy number-associated transcripts may have prognostic relevance and could aid in explaining some of the outcome variation among tumors of the same stage. Thus, although there may be some genes irrelevant to the cancer process differentially expressed due to a dosage effect of CNA, our data demonstrate that integration of genome CNA with expression in tumor cells with an aggressive phenotype (i.e. lymphotropism) could uncover candidate biomarkers or therapeutic targets. However, larger scale integrative analysis with more patient tissue samples is needed for further validation and new discoveries of DNA copy number-associated genes. In addition, functional studies are warranted to assess the biological roles of these DNA copy number driven genes in the OSCC progression.

## Conclusions

In this study, we have presented 1) an approach to systematically integrate DNA copy number aberrations and differential gene expression; 2) copy number-associated genes associated with OSCC status and survival; and 3) improved prognostication of 2-year survival using survival models based on copy number-associated transcripts compared to those using the current clinical standard, AJCC stage, alone. Further investigation is warranted to confirm these findings and examine the biologic role of these copy number-associated genes in OSCC metastasis and their potential as therapeutic targets.

## Methods

### Tissue collection and specimen processing

As described previously in Chen et al [21], we identified English-speaking patients 18 year of age or older with a first, primary OSCC at any of three University of Washington-affiliated hospitals: University of Washington Medical Center, Harborview Medical Center, and the Puget Sound Veterans Affairs Health Care System. Metastatic OSCC lymph nodes were snap-frozen at time of surgical resection and stored in liquid nitrogen until use. Tissue preparation, LCM of tumor cells, and extraction of DNA and RNA from the same LCM-harvested cell population was performed as previously described [32]. Purified RNA was stored in -80°C until use. Quantity and quality of purified RNA and DNA from each specimen was determined by ND-1000 spectrophotometer (Nano-Drop Technologies, Wilmington, DE) and Agilent 2100 bioanalyzer (Agilent Technologies, Santa Clara, CA).. Purified DNA was dried on a Savant DNA Speed Vac 110 (Global Medical Instrumentation, Ramsey, MN), re-dissolved as 50 ng/μl in 10 mM Tris-HCl pH 8 with 0.1 mM EDTA, and stored at -20°C until use [32].

### Genome profiling with SNP array

The DNA was hybridized onto Affymetrix Human Mapping 250 K Nsp I SNP array (Affymetrix Inc., Santa Clara, CA) following the manufacturer's instructions. Briefly, 250 ng of purified genomic DNA was digested with restriction enzyme Nsp I, ligated to an Nsp I adaptor, and PCR amplified. After purification, 90 μg of amplified DNA were fragmented, biotin-labeled, and hybridized to the SNP array. Intensity of the hybridized feature was acquired with Affymetrix GCOS (v1.4) software. SNP genotyping was performed with the Robust Linear Model with Mahalanobis distance classifier algorithm (BRLMM) in Affymetrix Genotyping Console (v3.0) software. Quality of the genome profiling process was assessed by the yield and length range of the amplified DNA, the length range of the fragmented DNA, and the SNP call rate following the manufacturer's instructions [32]. The SNP array results were normalized through median smoothing coupled with the Invariant Set Normalization method in dChip [33]. We took the log2 ratio between our normalized SNP array results and 2 which is the expected number of DNA copies in a normal cell and estimated the DNA copy number using the R package cghFlasso in reference to the Affymetrix 48 HapMap normal samples. cghFlasso infers the underlying piecewise linear DNA copy number profiles with fused lasso regression, and calls gains and losses by controlling the overall false discovery rate [34]. The location of each SNP probe set was mapped to the human genome (Build 36.1) using Affymetrix annotation file 'Mapping250K_Nsp.na26.annot.csv'. The genome DNA copy number of a transcript was calculated as: 1) the average genome DNA copy number inferred from all the SNPs within the transcript if the transcript overlapped with 5 or more SNPs; 2) the average of the genome DNA copy numbers inferred by the closest 5 SNPs within a 250 kb neighboring region of the transcript; or 3) the average of the genome DNA copy numbers inferred by all the SNPs within 250 kb of the transcript if there were fewer than 5 SNPs in this region.

### Transcriptome profiling

The RNA was hybridized onto Affymetrix Human Genome U133 Plus 2.0 array (Affymetrix Inc., Santa Clara, CA) as previously described [7]. Briefly, 40 ng of purified total RNA was amplified using Arcturus RiboAmp Plus 1.5-round kit (Molecular Devices) following the manufacturer's instructions. Amplified RNA was biotin-labeled and fragmented using Affymetrix GeneChip IVT Labeling kit and hybridized to the U133 Plus 2.0 array following manufacturer's protocol. Intensity of the hybridized feature was acquired with Affymetrix GCOS (v1.4) software. Quality of the transcriptome profiling process was assessed by the yield and length range of the amplified RNA, the length range of the fragmented RNA, the average background, the "Present" call rate, and the 3'/5' ratio for glyceraldehyde-3-phosphate dehydrogenase and ß-actin following the manufacturer's instructions [32]. Gene expression profiles were normalized using the R package gcRMA http://www.bioconductor.org.The location of each transcript measured by each probe set was mapped to the human genome (Build 36.1) using Affymetrix annotation file 'HG-U133_Plus_2.na27'.

### Transcript filtering

We eliminated transcripts which: 1) had no alignment information (e.g., control probes) or no unique alignment to the human genome; 2) were on the sex chromosome and mitochondrial DNA; 3) had no expression values greater than 3 on a log2 scale in at least 3 samples; and 4) had an inter-quartile range of expression less than 0.1 on a log2 scale [21].

### Analysis of the association between DNA copy number and gene expression

To ensure that the median of the expression data from any sample or transcript was around zero with the same variation across samples or transcripts, a second normalization was employed after transcript filtering using the following formula:

where Ỹ is the median of Ys and |Y| is the absolute value of Y. Our goal was to find genes with expression associated with DNA copy number. For this purpose, we employed robust regression to evaluate the correlation between gene expression and DNA copy number across OSCC samples. Compared to least square regression (LSR), robust regression analysis provides an alternative when fundamental assumptions are unfulfilled by the nature of the data. In our case, it was equality of the error variance along the predicted line that did not hold. We used rlm function from R package MASS, in which iteratively reweighted least square algorithm was employed to estimate parameters in the regression model. For each gene, we fitted the model:

where Yi was the gene expression and Xi was the DNA copy number estimated from the SNPs. Two-sided P-value for the test of null hypothesis β = 0 was calculated using the t-statistics from the robust regression with a degree of freedom (df) = N-2 where N is the number of samples.

### Enrichment analysis of DNA copy number-associated transcripts

We identified copy number-associated transcripts meeting a False Discovery Rate (FDR) of < 1% (n = 530 transcripts) to determine if these represented an enriched subset of transcripts associated with OSCC status or survival by using our previously published, independent dataset comprising of 167 OSCC primary tumors and 45 normal controls [21], from which we excluded 19 patients whose metastatic lymph nodes were used for generating the CNA-associated transcripts. (1 OSCC patient whose metastatic lymph nodes were used to identify CNA associated transcripts was not part of Chen et al. 2008.). The association between the expression of these transcripts and being a case in this independent dataset comprising of 148 OSCC cases and 45 controls was assessed using a linear regression implemented by GenePlus software [35,36]. To determine the association between expression and survival, we analyzed expression array results for the subset of the 148 OSCC patients for which we had at least four months of follow-up time to reduce the inclusion of deaths from patients due to co-morbidities rather than tumor biology (n = 132). The association between expression and survival was estimated using Cox-regression adjusting for age and sex. A dummy variable was created for sex. Then to determine whether our copy number-associated transcripts (n = 530) represented an enriched subset of transcripts associated with OSCC (vs. normal control), we compared the observed Z-score distribution of the 530 transcripts to the expected Z-score distribution from randomly selecting a subset of transcripts the same size one thousand times. This comparison of Z-score distributions was repeated for survival.

### Comparing survival prediction models with AJCC stage

Using the subset of copy number-associated transcripts which were found to be associated with either the OSCC status or survival in our enrichment analysis above, we determined whether a model which incorporates gene expression data predicts survival better than stage alone. We first reduced the dimensionality of the gene expression data by calculating the first two principal components summarizing the expression of a subset of copy number-associated transcripts which in our enrichment analysis were found to be either differentially expressed in OSCC vs. normal control (n = 122) or associated with survival (n = 27) using Matlab version R2006b. We decided to include the transcripts associated with OSCC (vs. normal control) in the survival prediction based on our previously reported finding that a group of 131 genes differentially expressed genes in OSCC identified OSCC patients at high risk for poor survival [22]. We fit five summary Cox models of OSCC-specific survival. The first model contained stage alone as a continuous predictor, the second contained the first and second principal components summarizing expression of the 122 transcripts, and the third model contained first and second principal components for the 27 transcripts. The fourth and fifth models included principal components for the 122 and 27 transcripts respectively along with stage. Risk scores were calculated using a jackknife leave-one-out analysis for 4 models: two models using the first two principal component scores from the two subsets of correlated transcripts ('122 PC' and '27 PC'); and two other models combining AJCC stage and the principal component scores from either of the two transcript subsets ('stage + 122 PC' and 'stage + 27 PC'). For each model and for AJCC stage, we then used an adapted Receiver Operating Characteristics (ROC) analysis [37] to construct ROC curves for predicting two year survival as previously described [22].

### Ingenuity Pathways Analysis

The lists of the 122 and 27 transcripts were analyzed using Ingenuity Pathway Analysis software (IPA, version 8.5, Ingenuity^® ^Systems, http://www.ingenuity.com Core Analysis. We used the default settings so the Functions/Pathways/Tox list Analyses were performed with "Ingenuity Knowledge Base (Genes Only)" as reference set and both direct and indirect relationships were considered in the Network Analysis.

## Competing interests

The authors declare that they have no competing interests.

## Authors' contributions

CX carried out sample processing; laser-capture microdissection; genome profiling; transcriptome profiling; Ingenuity Pathway Analysis; participated in data analysis design; and drafted the manuscript. YL carried out statistical analysis of estimating DNA copy number and its association with gene expression; identified associations between copy number-associated genes and OSCC status and clinical outcomes; and revised; and revised the manuscript. PW gave guidance on statistical methodologies and tools; provided insightful explanation of the analytical results and major revisions to the manuscript. WF carried out the gene expression analysis; principal component analysis; and participated in data analysis design and revision of the manuscript. TCR carried out survival prediction modelling and participated in revision of the manuscript. MPU provided pathological support and participated in revision of the manuscript. JRH performed sample management and sample processing; and participated in revision of the manuscript. PL carried out the extraction of clinical information and participated in revision of the manuscript. DRD prepared datasets and conducted statistical analyses of clinical and outcomes data. NDF carried out patient recruitment and sample collection; and participated in overall design. LPZ participated in overall design and provided statistical guidance to WF for analysis of the gene expression data. SMS provided epidemiological support, guidance to DRD, and participated in overall design, planning of the data analysis; and provided major revisions to the manuscript. CC, as the principal investigator of the parent study (NIH RO1CA095419-06A1), provided the biospecimens and study participants-associated relevant data that were obtained through in-person baseline and follow-up interviews and medical record abstraction; provided guidance to PL in medical chart abstraction of clinical information; provided guidance to JRH to carry out sample management and processing; participated in overall study design and data analysis design; and provided major revisions to the manuscript. EM carried out overall study design; coordination of collaborations; protocol development; sample processing; experimental design; transcriptome profiling; Ingenuity Pathway Analysis; and data analysis design; provided guidance to CX to execute study design and to PL in medical chart abstraction for clinical information; and drafted and provided major revisions to the manuscript. All authors read and approved the final manuscript.

## Supplementary Material

Additional file 1 **Table S1**. Age, gender, cancer site and stage of OSCC patients used to identify copy number associated gene-expression Click here for file

Additional file 2 **Figure S1**. Consensus plot of genome-wide copy number gains and losses in the 20 lymph node metastatic OSCC Click here for file

Additional file 3 **Table S2**. Transcripts* and the SNPs in their neighboring regions Click here for file

Additional file 4 **Table S3**. Transcripts differentially expressed in OSCC vs. controls Click here for file
