# Supplementary material for: Integrative analysis of DNA copy number and gene expression in metastatic oral squamous cell carcinoma identifies genes associated with poor survival
Source: Mol Cancer. 2010 Jun 11;9:143. doi: 10.1186/1476-4598-9-143 (PMC2893102; doi:10.1186/1476-4598-9-143)
Supplement: Additional file 1 — Table S1. Age, gender, cancer site and stage of OSCC patients used to identify copy number associated gene-expression [file 1476-4598-9-143-S1.DOC]

**Table S1. Characteristics of the OSCC patients used to identify copy number associated gene-expression**

| **Category** | **Number** | **Percentage** |
| --- | --- | --- |
| **Age** |  |  |
| <40 | 1 | 5% |
| 40-49 | 5 | 25% |
| 50-59 | 8 | 40% |
| 60-69 | 3 | 15% |
| 70-79 | 2 | 10% |
| >79 | 1 | 5% |
| **Gender** |  |  |
| Male | 13 | 65% |
| Female | 7 | 35% |
| **Site** |  |  |
| oral cavity | 12 | 60% |
| oropharynx | 8 | 40% |
| **AJCC stage** |  |  |
| I | 0 | 0% |
| II | 1 | 5% |
| III | 2 | 10% |
| IV | 17 | 85% |
| **Tumor Size** |  |  |
| T1 | 3 | 15% |
| T2 | 10 | 50% |
| T3 | 4 | 20% |
| T4 | 2 | 10% |
| TX | 1 | 5% |
| **Nodal Status** |  |  |
| N1 | 3 | 15% |
| N2 | 17 | 85% |
| **Vital status** |  |  |
| Alive | 11 | 55% |
| Dead-OSCC | 5 | 25% |
| Dead-non-OSCC | 2 | 10% |
| Dead-unknown cause | 2 | 10% |
