# Supplementary material for: Integrative analysis of DNA copy number and gene expression in metastatic oral squamous cell carcinoma identifies genes associated with poor survival
Source: Mol Cancer. 2010 Jun 11;9:143. doi: 10.1186/1476-4598-9-143 (PMC2893102; doi:10.1186/1476-4598-9-143)
Supplement: Additional file 2 — Figure S1. Consensus plot of genome-wide copy number gains and losses in the 20 lymph node metastatic OSCC [file 1476-4598-9-143-S2.PPT]

## Slide 1
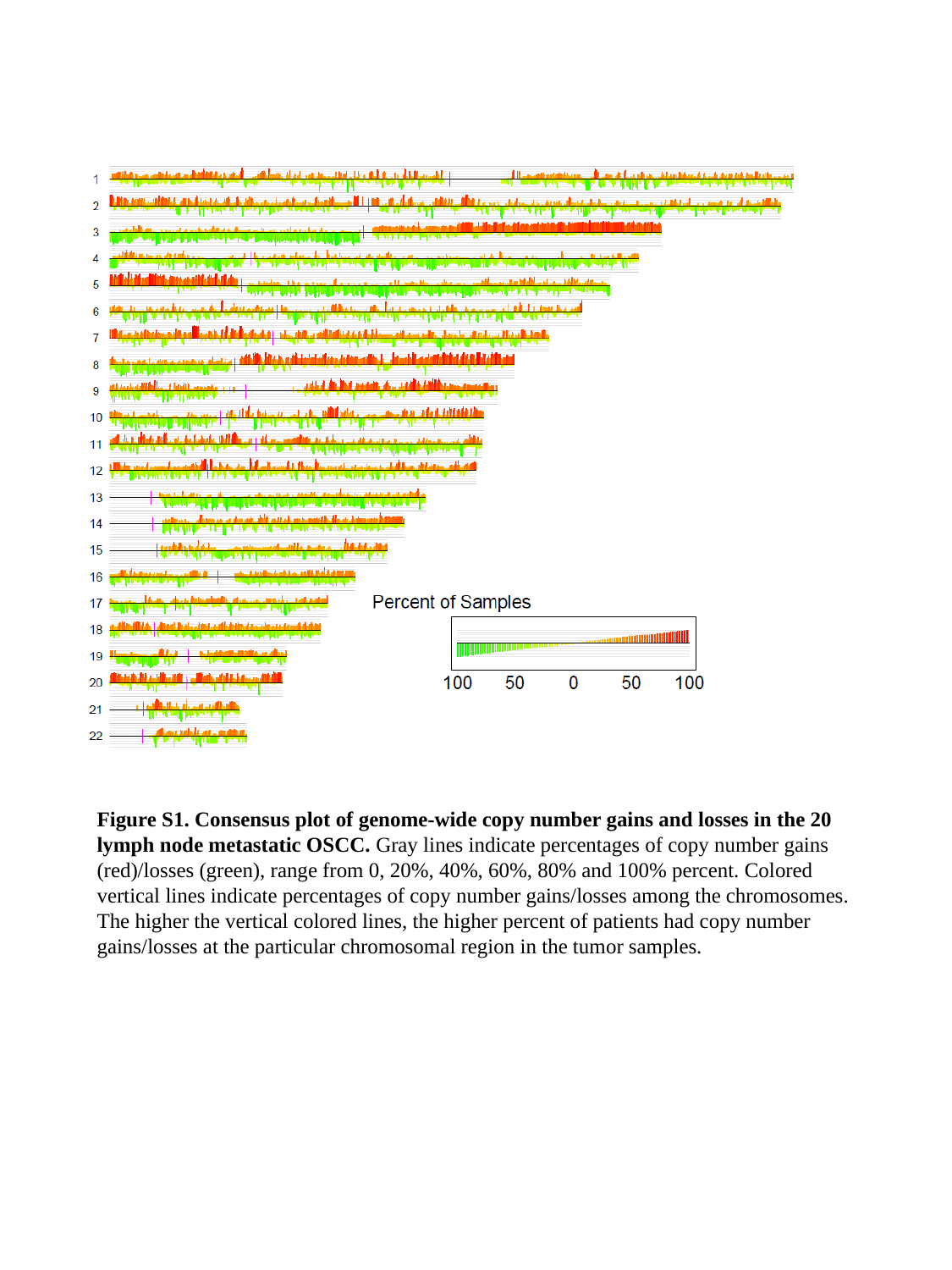

Figure S1. Consensus plot of genome-wide copy number gains and losses in the 20 lymph node metastatic OSCC. Gray lines indicate percentages of copy number gains (red)/losses (green), range from 0, 20%, 40%, 60%, 80% and 100% percent. Colored vertical lines indicate percentages of copy number gains/losses among the chromosomes. The higher the vertical colored lines, the higher percent of patients had copy number gains/losses at the particular chromosomal region in the tumor samples.
