# Supplementary material for: Integrative analysis of DNA copy number and gene expression in metastatic oral squamous cell carcinoma identifies genes associated with poor survival
Source: Mol Cancer. 2010 Jun 11;9:143. doi: 10.1186/1476-4598-9-143 (PMC2893102; doi:10.1186/1476-4598-9-143)
Supplement: Additional file 3 — Table S2. Transcripts* and the SNPs in their neighboring regions [file 1476-4598-9-143-S3.PPT]

## Slide 1
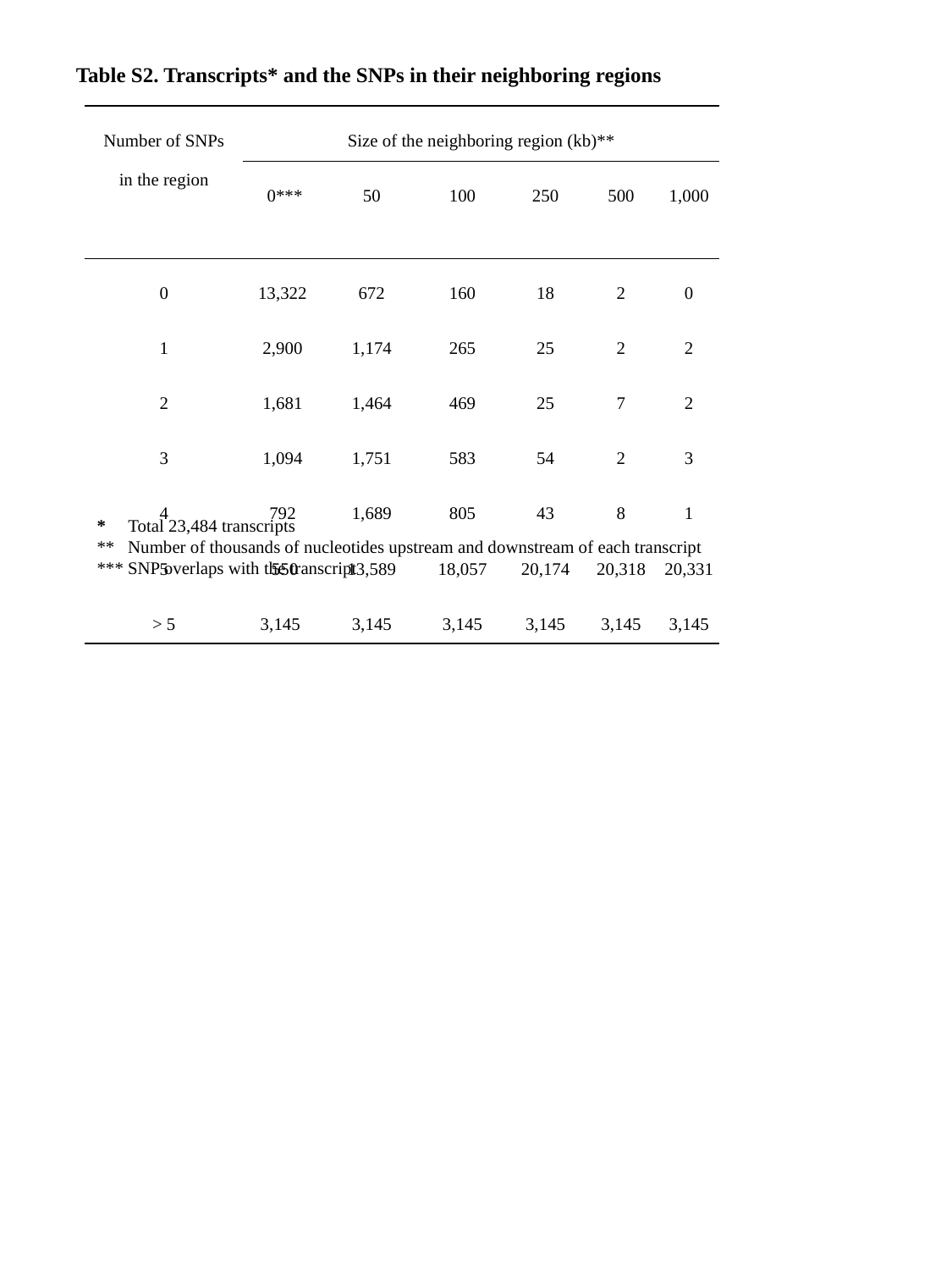

Table S2. Transcripts* and the SNPs in their neighboring regions
| Number of SNPs in the region | Size of the neighboring region (kb)\*\* | | | | | |
| --- | --- | --- | --- | --- | --- | --- |
| | 0\*\*\* | 50 | 100 | 250 | 500 | 1,000 |
| 0 | 13,322 | 672 | 160 | 18 | 2 | 0 |
| 1 | 2,900 | 1,174 | 265 | 25 | 2 | 2 |
| 2 | 1,681 | 1,464 | 469 | 25 | 7 | 2 |
| 3 | 1,094 | 1,751 | 583 | 54 | 2 | 3 |
| 4 | 792 | 1,689 | 805 | 43 | 8 | 1 |
| 5 | 550 | 13,589 | 18,057 | 20,174 | 20,318 | 20,331 |
| > 5 | 3,145 | 3,145 | 3,145 | 3,145 | 3,145 | 3,145 |
* Total 23,484 transcripts
** Number of thousands of nucleotides upstream and downstream of each transcript
*** SNP overlaps with the transcript
